# A Lipid-Structured Model of Atherosclerosis with Macrophage Proliferation

**DOI:** 10.1007/s11538-024-01333-w

**Published:** 2024-07-09

**Authors:** Keith L. Chambers, Michael G. Watson, Mary R. Myerscough

**Affiliations:** 1 School of Mathematics and Statistics, The University of Sydney, Sydney, NSW 2006 Australia; 2 Mathematical Institute, The University of Oxford, Oxford, Oxfordshire, OX2 6GG UK; 3 School of Mathematics and Statistics, The University of New South Wales, Sydney, NSW 2052 Australia

**Keywords:** Proliferation, Atherosclerosis, Non-local, Lipid, Structured

## Abstract

Atherosclerotic plaques are fatty deposits that form in the walls of major arteries and are one of the major causes of heart attacks and strokes. Macrophages are the main immune cells in plaques and macrophage dynamics influence whether plaques grow or regress. Macrophage proliferation is a key process in atherosclerosis, particularly in the development of mid-stage plaques, but very few mathematical models include proliferation. In this paper we reframe the lipid-structured model of Ford et al. (J Theor Biol 479:48–63, 2019. 10.1016/j.jtbi.2019.07.003) to account for macrophage proliferation. Proliferation is modelled as a non-local decrease in the lipid structural variable. Steady state analysis indicates that proliferation assists in reducing eventual necrotic core lipid content and spreads the lipid load of the macrophage population amongst the cells. The contribution of plaque macrophages from proliferation relative to recruitment from the bloodstream is also examined. The model suggests that a more proliferative plaque differs from an equivalent (defined as having the same lipid content and cell numbers) recruitment-dominant plaque in the way lipid is distributed amongst the macrophages. The macrophage lipid distribution of an equivalent proliferation-dominant plaque is less skewed and exhibits a local maximum near the endogenous lipid content.

## Introduction

Atherosclerosis is a chronic inflammatory disease of the artery wall (Back et al. 2019; Wolf and Ley 2019). The disease begins when disturbed blood flow allows fatty compounds called lipids, that are attached to low-density-lipoprotein (LDL) particles, to enter the artery wall from the bloodstream. The accumulation of LDL triggers an immune response which attracts circulating monocytes into the lesion. These monocytes rapidly differentiate into macrophages upon entry into the wall. Macrophages consume the LDL lipid in a process called *phagocytosis* and contribute to the further recruitment of monocyte-derived macrophages via inflammatory signalling (Moore et al. 2013; Xu et al. 2019). Over time the lesion may develop into an atherosclerotic plaque that contains a large necrotic core of extracellular lipids, due to the death of lipid-laden macrophages. The rupture of such a plaque releases the necrotic core material into the bloodstream, where it promotes blood clot formation. These blood clots can block vessels in the heart or the brain and induce myocardial infarction or stroke. The mechanisms of early atherosclerosis that lead to necrotic core formation remain an active research topic ( Gonzalez and Trigatti 2017).

Atherosclerotic plaque development is driven by the dynamic interaction between macrophages and lipids, in addition to the relative rates at which these constituents enter and leave the plaque (Moore et al. 2013). Macrophages ingest lipid by consuming nearby LDL particles, apoptotic (dying) cells and necrotic material ( Remmerie and Scott 2018; Baraniecki et al. 2023; Brouckaert et al. 2004). They can play a protective role by removing lipid from the plaque, either by offloading lipid to high-density lipoprotein (HDL) particles for transport out of the artery wall ( Groenen et al. 2021), or by emigrating from the plaque (Kang et al. 2021). However, if a macrophage undergoes apoptosis (programmed cell death) and is not efficiently cleared by a live macrophage (a process called *efferocytosis*), then the apoptotic cell will undergo secondary necrosis (Hou et al. 2023). Necrotic cell death causes the internal lipid of the macrophage to spill into the extracellular environment. This is how the necrotic core grows. The processes just described suggest that the number of plaque macrophages and the lipid ingested by these cells are likely to be influential factors in the determination of plaque fate. An additional, and often under-appreciated, mechanism that may substantially alter macrophage number and their quantity of internalised lipid is macrophage proliferation. This process is the focus of the current work.

Many plaque macrophages derive from the proliferation of macrophages that are already in the plaque; others are derived from monocytes recruited to the plaque from the bloodstream ( Lhoták et al. 2016; Robbins et al. 2013; Takahashi et al. 2002). However, the role of macrophage proliferation in plaque development is not well understood. Proliferation may play a protective role by increasing the overall number of plaque macrophages and hence increasing the capacity for necrotic core ingestion. However, proliferation may also serve as a significant source of plaque lipid. Scaglia et al. (2014) showed that intracellular lipid synthesis (fatty acids in particular) is required to complete cell division. These lipids are likely used to form the membrane of the daughter cells (Blank et al. 2017), and may contribute to the necrotic core upon secondary necrosis. Overall, it is not clear if macrophage proliferation is a net-protective effect (Kim et al. 2021; Xu et al. 2019). Hence it is not known whether therapies should promote or inhibit macrophage proliferation to reduce necrotic core growth. Mathematical modelling provides a means to explore this question.

Mathematical models of atherosclerosis that account for macrophage local proliferation are scarce. To the best of our knowledge, there are only two examples: a stochastic model by Simonetto et al. (2017), and a spatio-temporal PDE model by Mukherjee et al. (2020). Both models follow the established convention of partitioning the macrophage population into “macrophages" and “foam cells" ( Avgerinos and Neofytou 2019; Calvez et al. 2009; Chalmers et al. 2015, 2017) where foam cells are lipid-laden macrophages that take on a foamy appearance under the microscope. We argue that macrophage local proliferation cannot be faithfully represented in such a framework. The crux of the issue is what happens upon foam cell division. Upon division, the internalised lipid of a foam cell is divided amongst the two daughter cells. However, it is not clear how these daughter cells should be classed. If the foam cell is heavily lipid-laden, then the two daughter cells may contain enough lipid to both be classified as foam cells too. But if the foam cell contains only just enough lipid to meet the criterion to be classified as a foam cell, then the daughter cells will be classed as regular macrophages. The models of Mukherjee et al. (2020) and Simonetto et al. (2017) avoid this ambiguity by assuming that foam cells do not divide. However, this assumption is at odds with experimental evidence which indicates that macrophage local proliferation occurs at every level of lipid accumulation, at least in mice (Kim et al. 2018). A natural solution to the problem of foam cell division is to track the lipid content of the macrophages as a structural variable.

We use the model of Ford et al. (2019) for macrophage populations in atherosclerotic plaques as the foundation of this study on macrophage proliferation. This model uses the lipid that macrophages contain as a structural variable. This includes lipid from ingested LDL, from apoptotic cells and the endogenous lipid in macrophage membranes. The Ford model is a system of partial integro-differential equations which captures how the distribution of lipid in the live and apoptotic macrophage populations contributes to necrotic core formation. Monocyte recruitment is included via a boundary condition, onloading and offloading of lipid by continuous advection, efferocytosis via a non-local convolution integral, and apoptosis and emigration via kinetic terms. Steady state analysis of the Ford model indicates that there are two qualitatively distinct profiles of the macrophage lipid distribution. The results demonstrate the important role of emigration and efferocytosis in reducing eventual necrotic core lipid content. In this paper we modify the Ford model by adding proliferation. Mathematically, this introduces non-local terms to the set of integro-differential equations.

The remainder of this paper is structured as follows. Section 2 describes the extended model which accounts for macrophage proliferation. Proliferation is modelled using a pantograph-style source term and local sink term. Similar terms are found in cell-division studies for size-structured (Efendiev et al. 2018) and mass-structured models ( Sinko and Streifer 1971). Section 3 contains the results of the model analysis. This includes a numerical simulation of time-dependent solutions and an analytical steady state analysis. Finally, we discuss the implications of our results in Sect. 4.

## Methods

### Definitions

Let *m*(*a*, *t*) and *p*(*a*, *t*) be the number density of the live and apoptotic macrophages in the lesion respectively. These densities are measured with respect to lipid content $a \ge a_0$ and depend on time $t \ge 0$. The quantity $a_0$ denotes the endogenous lipid content contained in the macrophage cell membranes. The lipid content of the necrotic core is denoted by *N*(*t*). The total numbers of live and apoptotic macrophages in the lesion, denoted *M*(*t*) and *P*(*t*) respectively, are found by integrating over all possible lipid contents:

1 $$\begin{aligned} M(t) := \int _{a_0}^{\infty } m(a,t) da, \quad P(t) := \int _{a_0}^{\infty } p(a,t) da. \end{aligned}$$

Similarly, the total lipid contents of the live and apoptotic macrophage populations are given by:

2 $$\begin{aligned} A_M(t) := \int _{a_0}^{\infty } a m(a,t) da, \quad A_P(t) := \int _{a_0}^{\infty } a p(a,t) da. \end{aligned}$$

### Modelling Macrophage Local Proliferation

Our treatment of macrophage local proliferation is based on four assumptions:

1. **Macrophages divide at a constant rate**, $\rho$, **that is independent of the lipid content of the parent cell.**
  This assumption is made primarily for simplicity.
2. **Prior to division, the parent cell synthesises the additional** $a_0$ **lipid that is required to form two daughter cell membranes.**
  Here we account for experimental observations that synthesis of new lipid is required by the parent cell to form the membrane of the daughter cells (Scaglia et al. 2014; Rodriguez Sawicki et al. 2019). The exclusion of lipid synthesis from the model would render macrophages of lipid content $a < 2a_0$ unable to divide. We regard this as unphysical.
3. **Lipid synthesis and cell division occur on a faster timescale than macrophage population dynamics.**
  This is reasonable because mitosis typically lasts for 1 h (Araujo et al. 2016), whereas atherosclerosis progresses over months or years (Shah et al. 2015).
4. **Each cell division produces two daughter cells that contain an equal amount of lipid.**
  This final assumption is made primarily for simplicity but is expected to be a good approximation when averaging over many division events. Indeed, preferential sorting of phagocytic cargo into one daughter cell was only observed for infectious cargo in one study by Luo et al. (2008), and so it is reasonable to assume that non-infectious cargo such as lipids are evenly distributed upon division.

### Model Statement

The equations for our model are:

3 $$\begin{aligned}&\begin{aligned} \frac{\partial m}{\partial t} + \Big ( \frac{\lambda }{M} + \theta N \Big ) \frac{\partial m}{\partial a}&= \eta \int _{a_0}^{a-a_0} m(a-a', t) p(a', t) da' - \eta P m \\&\quad - (\beta + \gamma ) m + \underbrace{4\rho m( 2a - a_0, t) - \rho m}_{\text {local proliferation terms}}, \end{aligned} \end{aligned}$$

4 $$\begin{aligned}&\frac{\partial p}{\partial t} = \beta m - (\eta M + \nu ) p, \end{aligned}$$

5 $$\begin{aligned}&\frac{dN}{dt} = \nu A_P - \theta M N, \end{aligned}$$

where $a > a_0$ and $t > 0$. The above equations are coupled to the following boundary condition:

6 $$\begin{aligned} \Big ( \frac{\lambda }{M} + \theta N \Big ) m(a_0, t)&= \alpha \frac{A_M - a_0 M}{\kappa + A_M - a_0 M},&t&> 0. \end{aligned}$$

**Fig. 1:**
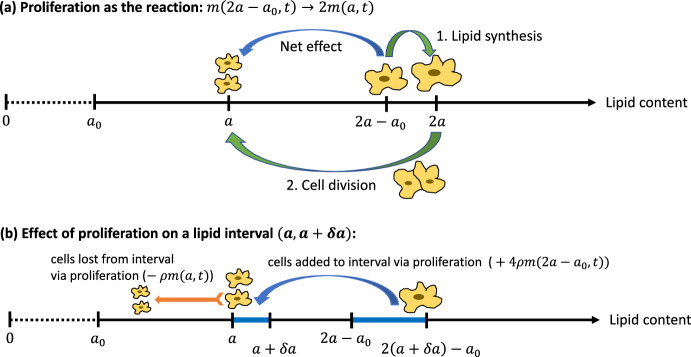
**a** Macrophage proliferation as the reaction $m(2a-a_0,t) \rightarrow 2m(a,t)$. During proliferation, lipid synthesis increases the lipid content of the cell by $a_0$. Synthesis is followed by cell division, which splits the total lipid equally into the two daughter cells. The net result is that macrophages of lipid content $2a - a_0$ proliferate into two cells of lipid content *a*. Both processes occur on a faster timescale than the population dynamics. **b** The source and sink processes, accounted for in Eq. (3), that arise from the reaction $m(2a-a_0,t) \rightarrow 2m(a,t)$

Macrophage local proliferation is treated in the final two terms of Eq. (3). As illustrated in Fig. 1, the source term, $4 \rho m (2a - a_0, t)$, accounts for macrophages of lipid content $2a - a_0$ proliferating into two macrophages of lipid content *a*. The factor $4 = 2^2$ is due to each division event producing two daughter cells, and the fact that macrophages proliferating into the infinitesimal interval $(a, a + \delta a)$ are sourced from the interval $(2a - a_0, 2a - a_0 + 2 \delta a)$, which is twice as large. The sink term, $- \rho m(a, t)$, accounts for macrophages of lipid load *a* dropping to a lower lipid content upon proliferation.

The remaining terms are from the original model of Ford et al. (2019), to which the interested reader is directed for detailed explanation. The advection term on the left side of Eq. (3) accounts for continuous changes in lipid content. This includes consumption of necrotic lipid (rate $\propto \theta$), and uptake of LDL and offloading to HDL at the net uptake rate $\lambda /M$. Here, the inverse proportionality to *M* arises from a quasi-steady approximation in the intraplaque dynamics of both LDL and HDL (see Ford et al. 2019 for a detailed derivation). By contrast, efferocytosis (rate $\propto \eta$) is modelled as a non-local increase in lipid content due to the consumption of whole apoptotic cells. The convolution source term in Eq. (3) accounts for all possible ways in which a macrophage can obtain a lipid content *a* following the consumption of an apoptotic cell. The integral is defined as 0 for $a < 2a_0$. The sink term $-\eta P m$ in Eq. (3) accounts for the loss of macrophages of lipid content *a* upon efferocytosis due to their non-local increase in lipid content. Macrophage uptake of apoptotic cells and necrotic lipid gives rise to further sink terms, $-\eta M p$ in Eq. (4) and $-\eta M N$ in Eq. (5), respectively. These terms are bilinear since uptake occurs in proportion to both the amount of macrophages and amount of apoptotic cells/ necrotic lipid in the lesion. The model also accounts for macrophage apoptosis (rate $\beta$), emigration from the lesion (rate $\gamma$) and the secondary necrosis of apoptotic cells (rate $\nu$). Finally, macrophage recruitment from the bloodstream is captured in the boundary condition (6). Recruited macrophages are assumed to carry only endogenous lipid, $a = a_0$. The rate of recruitment is an increasing function of the internalised lipid content of the live macrophage population, $A_M - a_0 M$. This reflects an assumption that the more lipid the macrophages carry, the more inflammatory cytokines they produce, and, hence, the more rapidly new macrophages are recruited to the plaque. Maximal recruitment occurs at rate $\alpha$ and half-maximal recruitment occurs when $A_M - a_0 M = \kappa$.

Additional ODEs for *M*(*t*) and *P*(*t*) can be derived by integrating Eqs. (3) and (4) with respect to $a \in (a_0, \infty )$. The dynamics of $A_M(t)$ and $A_P(t)$ can be found by multiplying (3) and (4) by *a* prior to integration. In this way, we obtain:

7 $$\begin{aligned} \frac{dM}{dt}&= \alpha \frac{A_M - a_0 M}{\kappa + A_M - a_0 M} + (\rho - \beta - \gamma ) M, \end{aligned}$$

8 $$\begin{aligned} \frac{dP}{dt}&= \beta M - (\eta M + \nu ) P, \end{aligned}$$

9 $$\begin{aligned} \frac{dA_M}{dt}&= \alpha a_0 \frac{A_M - a_0 M}{\kappa + A_M - a_0 M} + \rho a_0 M -(\beta + \gamma )A_M \nonumber \\&\quad + \lambda + \theta MN + \eta M A_P, \end{aligned}$$

10 $$\begin{aligned} \frac{dA_P}{dt}&= \beta A_M - (\eta M + \nu ) A_P. \end{aligned}$$

The Eqs. (5), (7)–(10) give a closed subsystem of ODEs for *M*(*t*), *P*(*t*), $A_M(t)$, $A_P(t)$ and *N*(*t*) that can be solved independently of the non-local PDE system (3)–(6). We note that proliferation appears explicitly in Eqs. (7) and (9), where it acts to increase *M*(*t*) due to cell division and $A_M(t)$ due to division-associated lipid synthesis.

#### Initial Conditions

Appropriate initial conditions must be prescribed in order to close the system of Eqs. (3)–(10). We set:

11 $$\begin{aligned} \begin{aligned} m(a,0) = m_0(a),&\quad p(a,0) = p_0(a), \\ M(0) = M_0, \, \, P(0) = P_0, \, \, A_M(0)&= A_{M0}, \, \, A_P(0) = A_{P0}, \, \, N(0) = N_0. \end{aligned} \end{aligned}$$

Here $M_0$, $P_0$, $A_{M0}$, $A_{P0}$ and $N_0$ are constants. The initial lipid distributions, $m_0(a)$ and $p_0(a)$, are assumed to be half-normal functions:

12 $$\begin{aligned} \frac{m_0(a)}{M_0} = \frac{p_0(a)}{P_0} = \frac{1}{a_\sigma } \sqrt{\frac{2}{\pi }} \exp \Big [ \frac{-(a-a_0)^2}{2 a_\sigma ^2} \Big ], \end{aligned}$$

with a common variance determined by the constant $a_\sigma > 0$. It follows that $A_{M0} = \int _{a_0}^\infty a m_0(a) da$ and $A_{P0} = \int _{a_0}^\infty a p_0(a) da$ satisfy the following relations:

13 $$\begin{aligned} A_{M0}&= M_0 \Big ( a_0 + \sqrt{\frac{2}{\pi }} a_\sigma \Big ),&A_{P0}&= P_0 \Big ( a_0 + \sqrt{\frac{2}{\pi }} a_\sigma \Big ), \end{aligned}$$

from which we derive the equation:

14 $$\begin{aligned} M_0 = \frac{\kappa \lambda \sqrt{2\pi }}{a_\sigma (\alpha \sqrt{2 \pi } a_\sigma - 2\lambda )}, \end{aligned}$$

by ensuring consistency with the boundary condition (6).

We note that biologically realistic initial conditions must satisfy $a_\sigma > \frac{\sqrt{2} \lambda }{\sqrt{\pi } \alpha }$ so that $M_0 > 0$. We further assume that the lesion is initially devoid of necrotic lipid: $N_0 = 0$, and contains fewer apoptotic cells than live cells: $P_0 < M_0$. To satisfy this last condition, we arbitrarily set $P_0 = 0.5 M_0$.

### Non-dimensionalisation

We scale macrophage lipid content in units of endogenous lipid, $a_0$, and time in units of mean macrophage lifespan, $\beta ^{-1}$, by setting:

15 $$\begin{aligned} \tilde{a}&:= a_0^{-1}a,&\tilde{t}&:= \beta t. \end{aligned}$$

The remaining variables are non-dimensionalised as follows:

16 $$\begin{aligned}&\tilde{m} (\tilde{a}, \tilde{t}) := \frac{a_0 m(a,t)}{M(t)}, \quad \tilde{p} (\tilde{a}, \tilde{t}) := \frac{a_0 p(a,t)}{P(t)}, \quad \tilde{M}(\tilde{t}) := \frac{\beta }{\alpha } M(t), \quad \tilde{P}(\tilde{t}) := \frac{\beta }{\alpha } P(t), \nonumber \\&\tilde{A}_M(\tilde{t}) := \frac{\beta }{a_0 \alpha } A_M(t), \quad \tilde{A}_P(\tilde{t}) := \frac{\beta }{a_0 \alpha } A_P(t), \quad \tilde{N}(\tilde{t}) := \frac{\beta }{a_0 \alpha } N(t). \end{aligned}$$

The scaling (16) ensures that

17 $$\begin{aligned} \int _1^\infty \tilde{m} (\tilde{a}, \tilde{t}) d\tilde{a} = \int _1^\infty \tilde{p} (\tilde{a}, \tilde{t}) d\tilde{a} = 1, \end{aligned}$$

and so $$\tilde{m} (\tilde{a}, \tilde{t})$$ and $$\tilde{p} (\tilde{a}, \tilde{t})$$ can be interpreted as probability distributions for the lipid contained in the live and apoptotic macrophage populations respectively. We use the same scale factor, $$\beta / \alpha $$, for the populations *M*(*t*) and *P*(*t*), and another common scale factor, $$\beta / a_0 \alpha $$, for the lipids $$A_M(t), A_P(t), $$ and *N*(*t*). This is a different nondimensionalisation to the one used in Ford et al. (2019) and is chosen to facilitate comparison between the variables during analysis.

**Table 1:** Non-dimensional parameters that appear in Eqs. (18)–(26)

Parameter	Definition	Interpretation
$\tilde{\rho }$	$\frac{\rho }{\beta }$	Dimensionless proliferation rate
$\tilde{\gamma }$	$\frac{\gamma }{\beta }$	Dimensionless emigration rate
$\tilde{\nu }$	$\frac{\nu }{\beta }$	Dimensionless secondary necrosis rate
$\tilde{\eta }$	$\frac{\alpha \eta }{\beta ^2}$	Dimensionless uptake rate of apoptotic lipid (efferocytosis)
$\tilde{\theta }$	$\frac{\alpha \theta }{\beta ^2}$	Dimensionless uptake rate of necrotic lipid
$\tilde{\lambda }$	$\frac{\lambda }{a_0 \alpha }$	Dimensionless net rate of lipid uptake via LDL/HDL
$\tilde{\kappa }$	$\frac{\kappa \beta }{a_0 \alpha }$	Dimensionless live cell accumulated lipid content for half-maximal recruitment
$\tilde{a}_\sigma$	$\frac{a_\sigma \beta }{a_0}$	Scale parameter that determines the spread of $\tilde{m}(\tilde{a}, 0).$

We also define a number of dimensionless parameters. These are listed in Table 1. The first three parameters, $\tilde{\rho }$, $\tilde{\gamma }$ and $\tilde{\nu }$ pertain to cellular kinetics. We note that the dimensionless proliferation rate, $\tilde{\rho }$, plays a central role in the present study. The next three parameters, $\tilde{\eta }$, $\tilde{\theta }$ and $\tilde{\lambda }$, can be interpreted as dimensionless rates of lipid uptake. The parameter $\tilde{\lambda }$ differs in form from $\tilde{\eta }$ and $\tilde{\theta }$, ultimately because the rate of lipid influx due to LDL uptake/HDL offloading is treated as constant while the rates of apoptotic and necrotic lipid uptake are proportional to $A_P$ and *N* respectively in Ford et al. (2019). The constant $\tilde{\kappa }$ is the (dimensionless) accumulated live lipid required for half-maximal macrophage recruitment, linking lipid uptake to the cellular kinetics. Finally, the scale factor $\tilde{a}_\sigma$ determines the spread of the initial distributions $\tilde{m}(\tilde{a}, 0) = \tilde{p}(\tilde{a},0)$.

By applying the scaling (16) and definitions of Table 1 to the model (3)–(10), and dropping the tildes for notational convenience, we obtain the following dimensionless PDEs:

18 $$\begin{aligned}&\begin{aligned} \frac{\partial m }{\partial t} + \Big ( \frac{\lambda }{M} + \theta N \Big ) \frac{\partial m}{\partial a}&= \eta P \Big [ \int _1^{a-1} m(a-a',t)p(a',t) da' - m(a,t) \Big ] \\&\quad +\rho \big [ 4 m(2a-1,t) - m(a,t) \big ] \\&\quad - \Big ( \frac{1}{M} \frac{A_M - M}{\kappa + A_M - M} + \rho \Big ) m(a,t), \end{aligned} \end{aligned}$$

19 $$\begin{aligned}&\frac{\partial p}{\partial t} = \frac{M}{P} \big [ m(a,t)-p(a,t) \big ], \end{aligned}$$

where the integral in Eq. (3) is defined as zero for $a < 2$. We also have the boundary condition:

20 $$\begin{aligned} \Big ( \frac{\lambda }{M} + \theta N \Big ) m(1,t)&= \frac{1}{M} \frac{A_M - M}{\kappa + A_M - M}, \end{aligned}$$

and the ODE system:

21 $$\begin{aligned} \frac{dM}{dt}&= \frac{A_M - M}{\kappa + A_M - M} + (\rho -1 -\gamma ) M, \end{aligned}$$

22 $$\begin{aligned} \frac{dP}{dt}&= M - (\eta M + \nu ) P, \end{aligned}$$

23 $$\begin{aligned} \frac{d A_M}{dt}&= \frac{A_M - M}{\kappa + A_M - M} + \rho M - (1 + \gamma ) A_M + \lambda + M (\eta A_P + \theta N), \end{aligned}$$

24 $$\begin{aligned} \frac{d A_P}{dt}&= A_M - (\eta M + \nu ) A_P, \end{aligned}$$

25 $$\begin{aligned} \frac{dN}{dt}&= \nu A_P - \theta M N. \end{aligned}$$

The model is closed with the initial conditions:

26 $$\begin{aligned} \begin{aligned}&m(a,0) = p(a,0) = \frac{2}{a_\sigma \sqrt{2\pi }} \exp \Big ( -\frac{(a-1)^2}{2 a_\sigma ^2} \Big ), \\&M(0) = 2P(0) = \frac{\kappa \lambda \sqrt{2 \pi }}{a_\sigma \big [ a_\sigma \sqrt{2 \pi } - 2\lambda \big ]}, \\&A_M(0) = 2 A_P(0) = \frac{\kappa \lambda \sqrt{2 \pi }}{a_\sigma \big [ a_\sigma \sqrt{2 \pi } - 2\lambda \big ]} \Big ( 1 + \frac{2}{\sqrt{2\pi }} a_\sigma \Big ), \\&N(0) = 0. \end{aligned} \end{aligned}$$

We note that time-dependent scaling of *m*(*a*, *t*) and *p*(*a*, *t*) in (16) gives rise to substantial differences between Eqs. (18), (19) and their respective dimensional equivalents (3), (4). In Eq. (18), the terms of the final line do not have equivalent counterparts in Eq. (3) and act to enforce the normalisation condition (17) by describing how the proportion of macrophages with lipid load *a* changes as a result of recruitment and proliferation. In Eq. (19), *m*(*a*, *t*) and *p*(*a*, *t*) now appear on the right with a common factor so that *p*(*a*, *t*) evolves at a rate proportional to the difference $m(a,t) - p(a,t)$.

### Model Parameterisation

The large parameter space (see Table 1) poses a significant challenge to our analysis of the model (18)–(26). Since the intention of the present study is to investigate the influence of proliferation on the model dynamics, we choose to fix the parameters that are peripheral to this purpose. Specifically, we take $\tilde{\nu } = 0.8$, $\tilde{\kappa } = 5$, $\tilde{a}_\sigma = 2$ and $\tilde{\lambda } = 0.1$.

The estimate for $\tilde{\nu } = \nu /\beta$ is based upon a dimensional apoptosis rate of $\beta = 0.05$ h$^{-1}$ and secondary necrosis rate of $\nu = 0.06$ h $^{-1}$. The stated apoptosis rate is the half-maximal value reported in the study of Thon et al. (2018), in which the authors obtain estimates by fitting their ODE model to *in vitro* data of lipid-laden macrophages. The secondary necrosis rate is derived from observations by Collins et al. (1997) that cell lysis occurs within 12–24 h following apoptosis.

It is likely that $\tilde{\kappa }$ and $\tilde{a}_\sigma$ are both order 1 quantities since $a_0$ is a natural unit for macrophage lipid content. We assume further that $\tilde{\kappa } > \tilde{a}_\sigma$ since otherwise there would be significant macrophage recruitment even in the absence of LDL influx. To arbitrarily satisfy this requirement, we take $\tilde{\kappa } = 5$ and $\tilde{a}_\sigma = 2$.

With $\tilde{\kappa }$ and $\tilde{a}_\sigma$ fixed, we see from Eq. (26) that the choice of initial conditions amounts to a specification of $\tilde{\lambda }$. We take $\tilde{\lambda } = 0.1$ so that the initial macrophage population is small: $M(0) \approx 0.13$. This is to model the presence of tissue-resident macrophages in the lesion prior to LDL infiltration at $t = 0$.

The remaining parameters, $\tilde{\rho }$, $\tilde{\gamma }$, $\tilde{\theta }$ and $\tilde{\eta }$ are considered over a range of values in our analysis. Although accurate estimation of these parameters is not currently feasible due to a lack of quantitative *in vivo* data, there are observations that inform our choice of these parameter values. Firstly, we assume that $\tilde{\eta } > \tilde{\theta }$ to be consistent with reports that the uptake of apoptotic cells is more efficient than uptake of necrotic material (Kojima et al. 2017). We note also that the dimensionless emigration rate is likely to take values near $\tilde{\gamma } \approx 0.25$. This is based on a dimensional emigration rate of $\gamma = 0.013$ h$^{-1}$, which is an intermediate value between the 12.6% transmigration rate reported in Angelovich et al. (2017) and 20 h residence time reported in Ghattas et al. (2013). Finally, we will assume throughout that $\tilde{\rho } < 1 + \tilde{\gamma }$. This is a restriction based upon Eq. (21), which predicts unbounded growth in *M*(*t*) if $\tilde{\rho } \ge 1 + \tilde{\gamma }$. Biologically, this regime corresponds to a scenario where the lesion macrophages proliferate faster than they leave the system via apoptosis or emigration.

### Numerical Solution Scheme

Numerical solutions for the Eqs. (18)–(26) are obtained by using the method of lines and integrating the resulting ODE system with the Wolfram Mathematica routine *NDSolve*. The semi-infinite *a*-domain ($a \ge 1$) is approximated by a large finite interval, $1 \le a \le a_{\times }$, and discretised uniformly. In Eq. (18), we approximate the lipid derivative $\frac{\partial m}{\partial a}$ using the second order upwinding scheme and the integral term via the trapezoidal rule. Finally, we note that the proliferation source term $4 \rho m(2a-1,t)$ is ill-defined for $2a-1 > a_{\times }$. We therefore omit this term in our numerical scheme when $a > \frac{a_{\times }+1}{2}$. This omission is justified for large $a_{\times }$ since *m*(*a*, *t*) is a probability function and must therefore satisfy: $m(a,t) \rightarrow 0$ as $a \rightarrow \infty$. In Appendix A, we use that $\int _1^\infty m(a,t) da = 1$ to quantify this error as a function of $a_\times$. We set $a_\times = 100$ for all numerical simulations in this paper. This value ensures that deviations from normalisation are typically smaller than $10^{-3}$.

## Results

### Time-Dependent Solutions

**Fig. 2:**
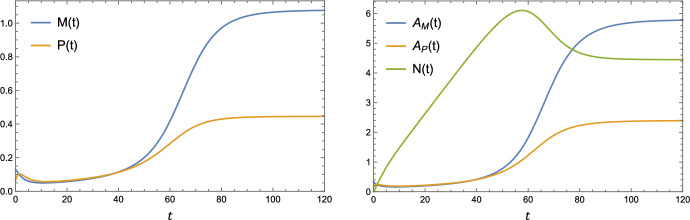
Time-dependent solutions for the ODE variables. The parameter values used are: $\rho = 0.8$, $\gamma = 0.25$, $\eta = 1.5$, $\theta = 0.4$

Typical time-dependent solutions for the ODE variables are shown in Fig. 2. The dynamics appear to transition through four distinct stages. The first stage, spanning $0 \le t \lessapprox 10$, is an initial transient in which *M*(*t*) decreases slightly due to a low rate of recruitment relative to loss via apoptosis and emigration. There is a corresponding initial increase in *P*(*t*), followed by the function peaking and decreasing due to secondary necrosis. The lipid quantities $A_M(t)$ and $A_P(t)$ exhibit similar behaviour to *M*(*t*) and *P*(*t*) respectively. There is near-linear growth in *N*(*t*) as the uptake rate of necrotic lipid (proportional to $\theta M(t)$) is small. In the second stage ($10 \lessapprox t \lessapprox 50$), the system exhibits a slow increase in *M*(*t*), *P*(*t*), $A_M(t)$ and $A_P(t)$. These trends reflect a macrophage population that is gradually accumulating lipid from LDL uptake (modelled with $\lambda > 0$) and endogenous lipid from efferocytosis. The necrotic core continues to grow in an approximately linear fashion. The dynamics enter a third stage when *N*(*t*) grows large enough that uptake of necrotic lipid becomes comparable to uptake of apoptotic lipid: $\theta N(t) \sim \eta A_P(t)$. This occurs at $t \approx 50$ for the parameter values used in Fig. 2. When the necrotic core becomes an additional substantial source of lipid uptake, this gives rise to a large increase in live lipid content, $A_M(t) - M(t)$ and a corresponding increase in the recruitment of live macrophages, *M*(*t*). The growth of *N*(*t*) slows before the function peaks and begins decreasing as *t* increases. Finally (stage 4), the system reaches an equilibrium at $t \approx 120$.

**Fig. 3:**
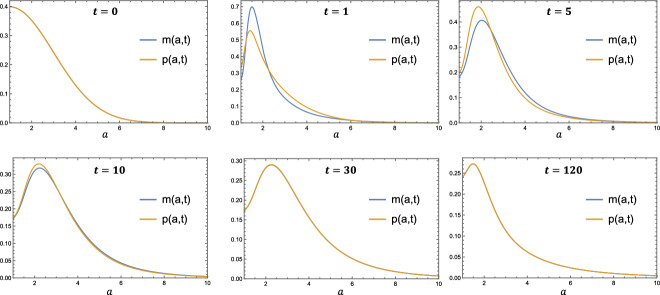
Time evolution of the lipid distributions *m*(*a*, *t*) and *p*(*a*, *t*). The parameter values used are: $\rho = 0.8$, $\gamma = 0.25$, $\eta = 1.5$, $\theta = 0.4$. Note that the curves *m*(*a*, *t*) and *p*(*a*, *t*) are concurrent in the first, fifth and sixth panes

The time evolution of the lipid distributions *m*(*a*, *t*) and *p*(*a*, *t*) is shown in Fig. 3. The simulation begins with $m(a,t) = p(a,t)$ as half-normal distributions, in accordance with the initial conditions (26). For very early times, $0 < t \lessapprox 1$, the live lipid distribution, *m*(*a*, *t*), becomes increasingly concentrated towards lower lipid loads. Since *m*(1, *t*) (which is proportional to the recruitment rate) decreases from its initial value, this early change is likely due to macrophage proliferation as opposed to recruitment. As *t* increases between $1 \lessapprox t \lessapprox 30$, lipid uptake drives a gradual increase in *m*(*a*, *t*) at large values of *a*, reflecting a growth in the proportion of highly lipid-laden macrophages. As the system approaches equilibrium near $t = 120$, *m*(*a*, *t*) becomes concentrated towards $a = 1$ once again. This final overall decrease in lipid load is most likely driven by increased macrophage recruitment, rather than proliferation, as can be seen by the increase in *m*(1, *t*). We note that *p*(*a*, *t*) and *m*(*a*, *t*) are close to equal, except at early but nonzero values of *t*. This is due to the form of Eq. (19) which states that *p*(*a*, *t*) evolves towards *m*(*a*, *t*) at a rate proportional to $\frac{M(t)}{P(t)}$. Since $\frac{M(t)}{P(t)}$ is an order 1 quantity throughout the simulation (see Fig. 2), the rapid early change in *m*(*a*, *t*) for $0 < t \lessapprox 1$ causes a difference between *m*(*a*, *t*) and *p*(*a*, *t*) early in the simulation. This difference is resolved on the order 1 timescale, with $m(a,t) \approx p(a,t)$ by $t = 10$.

Numerical simulations indicate that the system evolves towards a steady state, as indicated by Figs. 2 and 3. For the parameter values we considered, steady state is typically effectively attained within $t = 200$ macrophage lifetimes, corresponding to 167 days. Atherosclerotic plaques can exist for decades in humans, and so the steady state provides biologically useful information ( Kusuma Venkatesh and Venkatesha 2018).

### Steady State Analysis: ODE Subsystem

Let $M^\star$, $P^\star$, $A_M^\star$, $A_P^\star$ and $N^\star$ denote the steady values of the ODE variables. These constants can be found by setting the time derivatives in Eqs. (21)–(25) to zero and solving the resulting system of five algebraic equations. Upon doing so, we find that each of the steady values can be expressed in terms of $M^\star$:

27 $$\begin{aligned} \begin{aligned} P^\star&= \frac{M^\star }{\eta M^\star + \nu },&A_M^\star&= M^\star + \kappa \Big ( \frac{1}{1- ( 1 +\gamma - \rho ) M^\star } -1 \Big ), \\ A_P^\star&= \frac{A_M^\star }{\eta M^\star + \nu },&N^\star&= \frac{\nu A_P^\star }{\theta M^\star }, \end{aligned} \end{aligned}$$

and that $M^\star$ itself satisfies the quadratic equation:

28 $$\begin{aligned} (1 + \gamma - \rho ) (M^\star )^2 + \big [ \lambda -1 + \gamma (\kappa + \gamma \kappa + \lambda ) - \rho (\lambda + \gamma \kappa ) \big ] M^\star - \lambda = 0. \end{aligned}$$

Only one of the two possible solutions to Eq. (28) corresponds to a valid steady state. This can be seen by considering the product of the roots: $\frac{- \lambda }{ 1 + \gamma - \rho }$. Since $\rho < 1 + \gamma$ (see Sect. 2.5), the product of roots is negative and it follows that there is only one positive solution:

29 $$\begin{aligned} \begin{aligned} M^\star&= - \frac{\lambda -1 + \gamma (\kappa + \gamma \kappa + \lambda ) - \rho (\lambda + \gamma \kappa )}{2(1+\gamma - \rho )} \\&\quad + \frac{\sqrt{\big [ \lambda -1 + \gamma (\kappa + \gamma \kappa + \lambda ) - \rho (\lambda + \gamma \kappa ) \big ]^2 + 4 \lambda (1 + \gamma - \rho )}}{2(1+\gamma - \rho )}. \end{aligned} \end{aligned}$$

It can be shown by substituting the solution (29) into the relations (27) that $A_M^\star > M^\star$, $A_P^\star > P^\star$ and $N^\star > 0$. Hence, a unique and valid steady state always exists for the model, and is given by Eq. (29). Although it is difficult to prove, numerical simulations of the ODE subsystem indicate that the steady state is indeed stable.

#### Influence of Proliferation on the ODE Steady State

The steady values of the ODE variables change monotonically as the proliferation parameter, $\rho$, is increased from 0 to $1 + \gamma$. We prove this result in Appendix B by taking partial derivatives of the equilibrium values with respect to $\rho$. Explicitly, we find that $M^\star$, $P^\star$ and $A_M^\star$ are increasing functions of $\rho$, that $N^\star$ is a decreasing function of $\rho$, and that $A_P^\star$ can be either monotone increasing or decreasing with $\rho$ depending on whether $\eta < \frac{(1+\gamma ) \nu }{\lambda }$ or $\eta > \frac{(1+\gamma ) \nu }{\lambda }$ respectively. Moreover, we can use Eqs. (27) and (29) to explicitly derive the limiting values of the ODE steady state variables with respect to $\rho$. Explicitly, we find the following limits as $\rho \rightarrow (1+\gamma )^{-}$:

30 $$\begin{aligned} M^\star , A_M^\star \rightarrow \infty , \quad P^\star \rightarrow \frac{1}{\eta }, \quad A_P^\star \rightarrow \frac{1+\gamma }{\gamma \eta }, \qquad \text {and } \quad N^\star \rightarrow 0. \end{aligned}$$

**Fig. 4:**
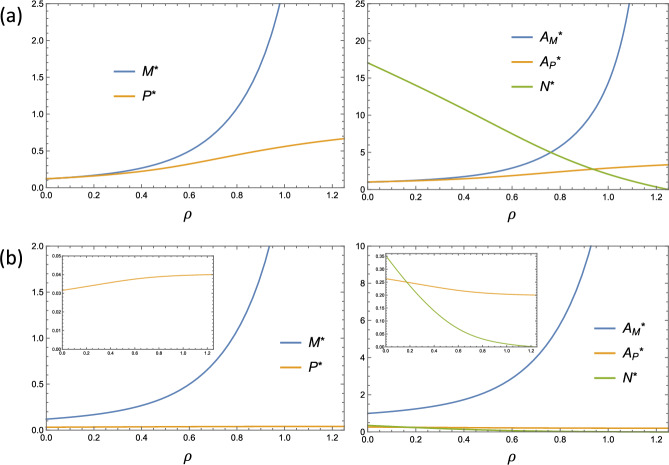
Dependence of the ODE equilibrium values on the proliferation rate, $\rho$. The model output for two sets of parameter values is shown, labelled (**a** and **b**). Set **a** uses $\gamma = 0.25$, $\eta = 1.5$, $\theta = 0.4$ so that $\eta < \frac{(1+\gamma ) \nu }{\lambda }$. Set **b** uses higher cellular lipid uptake rates: $\gamma = 0.25$, $\eta = 25$, $\theta = 5$ so that $\eta > \frac{(1+\gamma ) \nu }{\lambda }$. The two sets exhibit opposing trends for $A_P^\star$

Hence, the model predicts that a large enough proliferation rate is always capable of eliminating the necrotic core, albeit at the expense of having an extremely large cell population in the lesion. Plots of the ODE equilibrium values against $\rho$ are given in Fig. 4.

The result that $M^\star$ and $P^\star$ are increasing functions of $\rho$ is unsurprising since cell division directly increases the number of cells in the lesion. It is also expected that $A_M^\star$ increases with $\rho$ due to the lipid synthesis that occurs with proliferation. Interestingly, the results indicate that when the efferocytosis rate is small enough so that $\eta < \frac{(1+\gamma ) \nu }{\lambda }$, increases in the proliferation rate give rise to a smaller necrotic core, $N^\star$, despite increasing the amount of apoptotic lipid in the lesion, $A_P^\star$. In this case, the reduction in necrotic core lipid content is therefore conclusively due to an increase in the consumption of necrotic lipid by the live macrophage population. When $\eta > \frac{(1+\gamma ) \nu }{\lambda }$, increases to the proliferation rate also decrease the eventual necrotic core lipid content by reducing the amount of apoptotic lipid in the lesion (which sources the necrotic core via secondary necrosis). However, as indicated in Fig. 4b, the reduction in $A_P^\star$ is minimal in comparison to the increase in necrotic lipid uptake, which is proportional to $\theta M^\star$. Hence, in both cases the primary mechanism by which increases in the proliferation rate reduce eventual necrotic core lipid content is by increasing the total rate of necrotic lipid consumption at equilibrium.

**Fig. 5:**
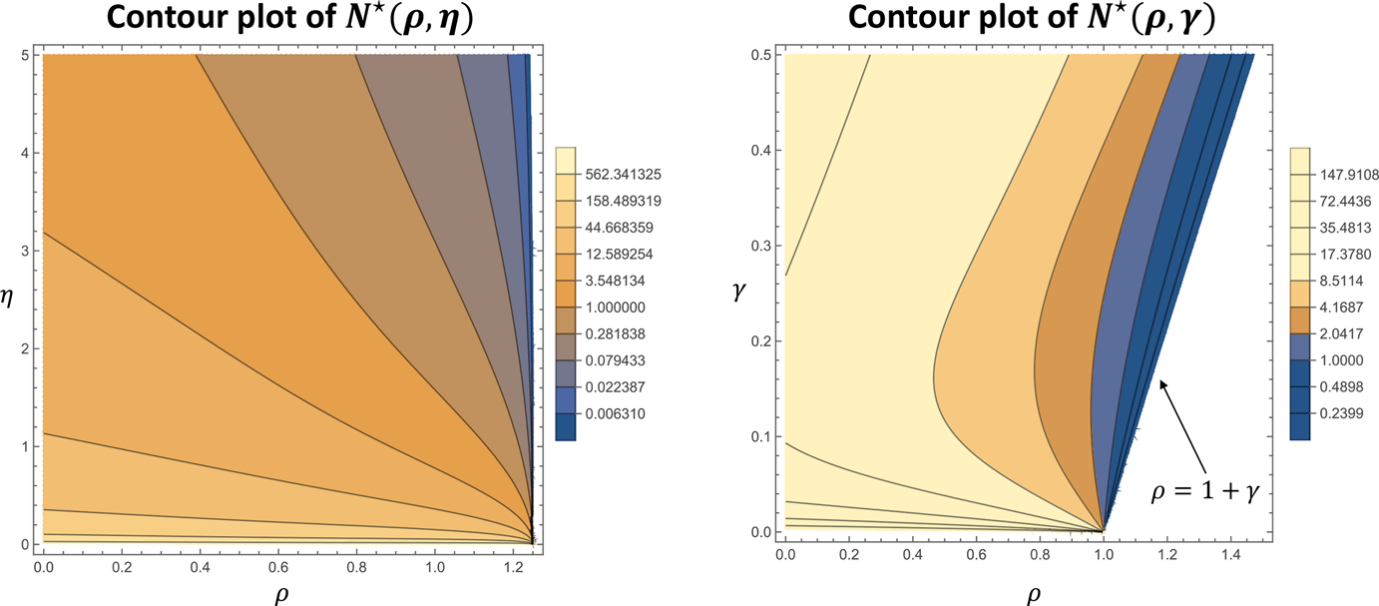
Contour plots of the equilibrium necrotic core lipid content, $N^\star$. The left plot illustrates the dependence on $\rho$ and $\eta$, the rates of proliferation and efferocytosis respectively. We take $\theta = \frac{\eta }{2}$ to ensure that $\theta < \eta$ (see Sect. 3.2.1) and set $\gamma = 0.25$. The right plot shows the dependence on $\rho$ and the emigration rate, $\gamma$. We use $\eta = 1.5$ and $\theta = 0.4$. The equilibrium only exists for $\rho < 1 + \gamma$, as indicated. Note that we use a logarithmic scale for the contours of both plots since $N^\star$ varies over a large range of values

To better illustrate the role of proliferation in determining the equilibrium necrotic core lipid content, $N^\star$, we vary $\rho$ with other model parameters simultaneously in Fig. 5. Consider firstly the left plot, in which $N^\star$ is plotted as a function of $(\rho , \eta )$. Here we also take $\theta = \frac{\eta }{2}$ to arbitrarily satisfy the requirement that $\eta > \theta$ (see Sect. 3.2.1). The plot indicates that increases in the proliferation rate reduce necrotic core lipid content most effectively when $\eta$ is large (where the contours are more vertical). Indeed when $\eta$ is small the contours are approximately horizontal. Hence, increases in the proliferation rate are ineffective at decreasing necrotic core lipid content when efferocytosis is defective. The right hand plot in Fig. 5, where $N^\star$ is plotted as a function of $\rho$ and $\gamma$, shows a non-monotone dependence of $N^\star$ on $\gamma$ for fixed $\rho < 1$. This indicates that there is a nonzero emigration rate ($\gamma \approx 0.16$ in the figure) for which $N^\star$ is minimised for given $\rho$. Furthermore, the plot indicates that $N^\star$ monotonically increases with $\gamma$ for $\rho > 1$. This result suggests that increasing the migratory propensity of a self-replenishing lesion macrophage population will increase necrotic core lipid content, probably because macrophages leave before they ingest much lipid.

### Steady State Analysis: *m*(*a*, *t*) and *p*(*a*, *t*)

Setting the time derivative to zero in Eq. (19) gives $p = m$. Hence, the live and apoptotic macrophages have identical lipid distributions at steady state. It then follows from Eqs. (18) and (20) that the equilibrium live macrophage lipid distribution, $m^\star (a):= \lim _{t \rightarrow \infty } m(a,t)$, satisfies the following boundary value problem:

31 $$\begin{aligned} \frac{d m^\star }{d a}&= k_1 \int _1^{a-1} m^\star (a-a') m^\star (a') da' - k_2 m^\star (a) + k_3 m^\star (2a-1), \end{aligned}$$

32 $$\begin{aligned} m^\star (1)&= m^\star _1, \end{aligned}$$

where the integral in Eq. (31) is defined as zero for $a < 2$, and $k_1$, $k_2$, $k_3$ and $m^\star _1$ are constants defined in terms of the ODE equilibrium values:

33 $$\begin{aligned} k_1&= \Big ( \frac{\lambda }{M^\star } + \theta N^\star \Big )^{-1} \eta P^\star ,&k_2&= \Big ( \frac{\lambda }{M^\star } + \theta N^\star \Big )^{-1} (\eta P^\star + 1 + \gamma + \rho ), \end{aligned}$$

34 $$\begin{aligned} k_3&= 4 \Big ( \frac{\lambda }{M^\star } + \theta N^\star \Big )^{-1} \rho ,&m^\star _1&= \Big ( \frac{\lambda }{M^\star } + \theta N^\star \Big )^{-1} \frac{1}{M^\star } \frac{A^\star _M - M^\star }{\kappa + A_M^\star - M^\star }. \end{aligned}$$

The combined delay and advanced dependence on *a* in Eq. (31) makes it difficult to find a general formula for $m^\star (a)$. The case $k_3 = 0$ (no proliferation) is solved in Ford et al. (2019) by partitioning the domain $a \ge 1$ into unit intervals and solving successively on each interval. An analytical solution can also be found for the case $k_1 = 0$ (no efferocytosis) using the Laplace transform. It is given by:

35 $$\begin{aligned} m^\star (a) = m^\star _1 \sum _{j = 0}^\infty \Big ( \frac{k_3}{2k_2} \Big )^j \sum _{i = 0}^j \frac{2^i}{\prod _{\ell = 0, \ell \ne i}^j (1-2^{i-\ell })} e^{-2^i k_2 (a-1)}. \end{aligned}$$

Our method, presented in Appendix C, is inspired by Hall and Wake (1989), who solved a similar problem in a model for cell growth. Unfortunately the solution (35) is too complicated to be biologically insightful and cannot, to the best of our knowledge, be manipulated into a closed-form expression.

Although a closed-form solution to Eq. (31) is unlikely to exist in full generality, we show below that the standard summary statistics do have closed-form expressions.

#### Summary Statistics for $m^\star (a)$

The statistical moments of $m^\star (a)$ are given by:

36 $$\begin{aligned} \varphi _n := \int _1^\infty a^n m^\star (a) da, \qquad n = 0, 1, 2, \dots . \end{aligned}$$

Our non-dimensionalisation (16) ensures that $\varphi _0 = 1$ and $\varphi _1 = \frac{A_M^\star }{M^\star }$. Analytical expressions for the higher order moments can be found recursively by multiplying Eq. (31) by $a^n$ and integrating over $a \in (1, \infty )$. Doing so and solving for $\varphi _n$ gives the full-history recurrence:

37 $$\begin{aligned} \varphi _n = \frac{m^\star _1 + n \varphi _{n-1} + k_3/2^{n+1} + \sum _{j=1}^{n-1} \left( {\begin{array}{c}n\\ j\end{array}}\right) \varphi _j (k_1 \varphi _{n-j} + k_3/2^{n+1})}{k_2 - 2k_1 - k_3/2^{n+1}}, \end{aligned}$$

for each $n \ge 2$. Using the recursion (37), closed-form expressions can be obtained for the standard summary statistics of a continuous probability distribution. For our analysis of $m^\star (a)$, we will use the mean, $\mu$, and skewness, $\tilde{\mu }_3$, defined by:

38 $$\begin{aligned} \mu&= \varphi _1,&\tilde{\mu }_3 = \frac{\varphi _3 - 3 \varphi _1 (\varphi _2 - \varphi _1^2)-\varphi _1^3}{(\varphi _2 - \varphi _1^2)^{3/2}}. \end{aligned}$$

Note that we do not explicitly consider the standard deviation of $m^\star (a)$ in the analysis below since the distribution is highly asymmetric.

#### Influence of Proliferation on $m^\star (a)$

**Fig. 6:**
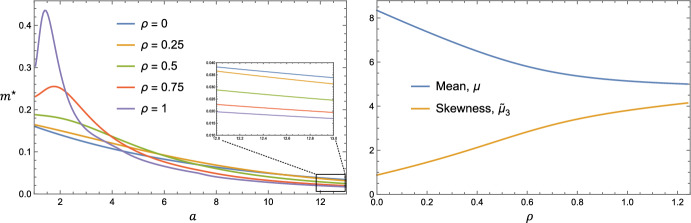
Influence of the proliferation rate, $\rho$, on the equilibrium lipid distribution, $m^\star (a)$. The left plot shows $m^\star (a)$ for various values of $\rho$. The right plot shows the corresponding summary statistics, computed using the formulae (38). The parameter values are set to: $\gamma = 0.25$, $\eta = 1.5$ and $\theta = 0.4$

Plots of $m^\star (a)$ for various values of $\rho$ are shown in Fig. 6. These solutions were obtained by numerically simulating the time-dependent system (18)–(26) to $t = 200$. The mean and skewness are also plotted as functions of $\rho$. We find that increasing $\rho$ reduces the average lipid burden per macrophage, $\mu$, and increases the skewness, $\tilde{\mu }_3$. Correspondingly, as $\rho$ increases, the proportion of macrophages with high lipid loads decreases and the proportion of macrophages with low lipid loads increases. Interestingly, we see that a local maximum develops near $a = 1$ for $\rho$ large enough.

**Fig. 7:**
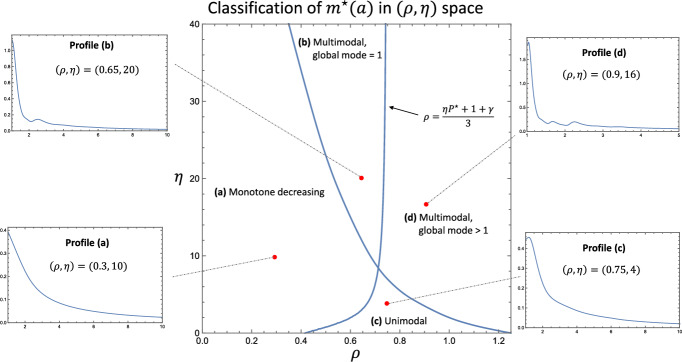
The four qualitatively distinct profiles of $m^\star (a)$ can be resolved in the $(\rho , \eta )$ subspace. In the central diagram, the curve separating regions (**a**, **b**) from (**c**, **d**) was is given by $\rho = \frac{\eta P^\star + 1 + \gamma }{3}$, and was plotted using the ODE equilibrium solutions (27) and (29). The curve separating regions **a** and **c** from **b** and **d** was found numerically by collecting 20 sample points and linking with second order interpolation. Examples for each profile are shown, using the $(\rho , \eta )$ values indicated by the red points. The remaining parameters are set to: $\gamma = 0.25$ and $\theta = 0.4$

The model exhibits four qualitatively distinct profiles for $m^\star (a)$. These can be resolved in the $(\rho , \eta )$ parameter-subspace, as seen in Fig. 7. If $\rho$ is small enough (quantified below), then $m^\star (a)$ either decreases monotonically, profile (a), or contains a series of peaks that are spaced approximately an integer apart, profile (b). These profiles are also observed in Ford et al. (2019). For $\rho$ large enough, the global maximum shifts from $a = 1$ to a local maximum at some $a_\text {max} > 1$. The precise condition that must be satisfied for this to occur is: $\rho > \frac{\eta P^\star + 1 + \gamma }{3}$, which is found by setting $\frac{d m^\star }{d a} \big \vert _{a = 1} > 0$ in Eq. (31). When the above condition holds, small values of $\eta$ result in a unimodal profile for $m^\star (a)$, profile (c). As $\eta$ is increased, a smaller secondary peak appears at $2 a_\text {max}$ due to macrophages containing $a_\text {max}$ lipid consuming equally lipid-laden apoptotic cells, profile (d). As seen in the figure, a tertiary peak can also be seen in some cases at $(2 a_\text {max} + 1)/2$ due to the proliferation of macrophages in the secondary peak.

**Fig. 8:**
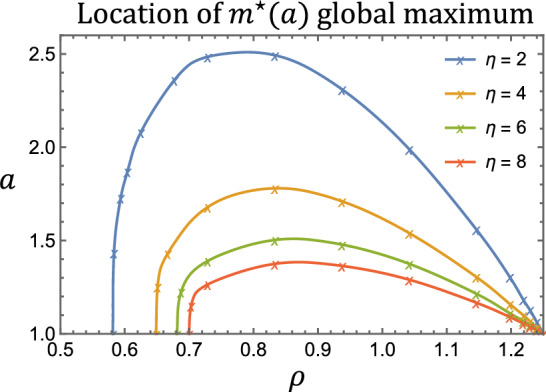
The value of *a* for which $m^\star (a)$ is maximised is sensitive to $\rho$ and $\eta$. We numerically computed the location of the maximum at the indicated values and linked the points with second order interpolation. The global maximum occurs at $a = 1$ for values of $\rho$ smaller than the plotted curves. The remaining parameters are set to: $\gamma = 0.25$ and $\theta = 0.4$

We also investigated the impact of $\rho$ and $\eta$ on the location of the global maximum of $m^\star (a)$ in Fig. 8. Consistent with Fig. 7, we find that the global maximum occurs at a value $a>1$ only when $\rho$ is sufficiently large. The smallest proliferation rate required for this behaviour, $\rho = \frac{\eta P^\star + 1 + \gamma }{3}$, increases with $\eta$. As $\rho$ increases, the global maximum location moves first to larger values of $a>1$, and then decreases to $a = 1$ as $\rho$ approaches its limiting value of $1 + \gamma$. The initial increase with $\rho$ occurs because proliferation promotes the accumulation of macrophages with a small amount of ingested lipid. This causes the global maximum location to shift from $a = 1$ to slightly higher values. The decrease to $a = 1$ for large proliferation rates occurs because macrophages are dividing so frequently in this limit that they cannot accumulate substantial lipid loads.

### Proliferation Versus Monocyte Recruitment

Macrophages in early atherosclerosis derive from either bloodstream recruitment or local proliferation (that is, proliferation in the artery wall). In this subsection, we use our model to explore how the relative contribution of these two processes impacts the eventual constitution of the plaque. Since the recruitment parameter, $\alpha$, appears throughout our non-dimensionalisation (16), we find it more convenient to work with dimensional quantities in the paragraphs below. These are adorned with a hat to avoid confusion.

We investigate the relative contribution of proliferation and recruitment by considering changes in $\hat{\rho }$ (the proliferation rate) and $\hat{\alpha }$ (proportional to the recruitment rate). As a control, we assume that $\hat{\rho }$ and $\hat{\alpha }$ are changed in such a way that the number of live macrophages in the plaque at steady state, $\hat{M}^\star$, remains fixed. All other parameters are also held constant. To derive the algebraic constraint that is imposed on $\hat{\rho }$ and $\hat{\alpha }$ by the above conditions, we set $\hat{M^\star }_{\hat{\alpha } = \hat{\alpha }_1, \hat{\rho } = \hat{\rho }_1} = \hat{M^\star }_{\hat{\alpha } = \hat{\alpha }_2, \hat{\rho } = \hat{\rho }_2}$ and expand using the dimensional form of Eq. (29). The equation reduces to

39 $$\begin{aligned} \frac{\hat{\beta } + \hat{\gamma } - \hat{\rho }_1}{\hat{\alpha }_1} = \frac{\hat{\beta } + \hat{\gamma } - \hat{\rho }_2}{\hat{\alpha }_2}, \end{aligned}$$

revealing that $\hat{\rho }$ and $\hat{\alpha }$ must be adjusted such that the ratio $\frac{\hat{\beta } + \hat{\gamma } - \hat{\rho }}{\hat{\alpha }}$ is fixed.

When $\tilde{\rho }$ and $\tilde{\alpha }$ are changed according to the constraint (39), we find that there is no change in the total apoptotic population, $\hat{P}^\star$, live or apoptotic lipid totals, $\hat{A}_M^\star$, $\hat{A}_P^\star$, or necrotic core lipid content, $\hat{N}^\star$. Hence, the model predicts that these quantities are independent of the relative contribution of proliferation and recruitment, in the sense described above. This result can be seen analytically by considering the dimensional versions of the steady state solutions (27):

40 $$\begin{aligned} \begin{aligned} \hat{P}^\star&= \frac{\hat{\beta } \hat{M}^\star }{\hat{\eta } \hat{M}^\star + \hat{\nu }},&\hat{A}_M^\star&= \hat{a}_0 \hat{M}^\star + \hat{a}_0 \hat{\kappa } \frac{\big ( 1 + \frac{\hat{\gamma }}{\hat{\beta }} - \frac{\hat{\rho }}{\hat{\beta }} \big ) \hat{M}^\star }{1 - \frac{\hat{\beta } + \hat{\gamma } - \hat{\rho }}{\hat{\alpha }} \hat{M}^\star }, \\ \hat{A}_P^\star&= \frac{\beta \hat{A}_M^\star }{ \hat{\eta } \hat{M}^\star + \hat{\nu }},&\hat{N}^\star&= \frac{\hat{\nu } \hat{A}_P^\star }{ \hat{\theta } \hat{M}^\star }. \end{aligned} \end{aligned}$$

Since $\hat{M}^\star$ and $\frac{\hat{\beta } + \hat{\gamma } - \hat{\rho }}{\hat{\alpha }}$ are fixed quantities, it follows that $\hat{P}^\star$ and $A_M^\star$ are also fixed. The same can then be concluded for $\hat{A}_P^\star$ and $\hat{N}^\star$, which depend on $\tilde{A}_M^\star$ and $\tilde{A}_P^\star$ respectively.

**Fig. 9:**
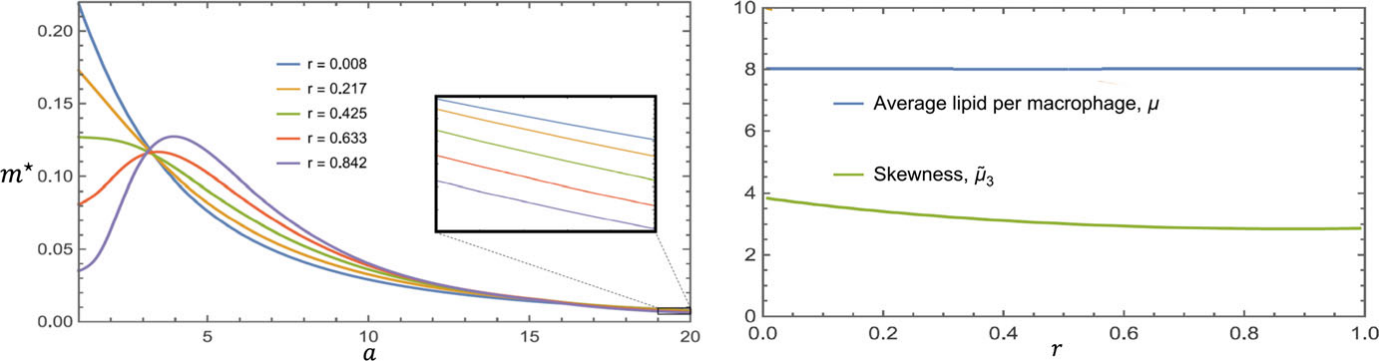
Changes in the relative contribution of proliferation and recruitment affect $m^\star (a)$. The left plot shows $m^\star (a)$ for various values of *r*. We note that the curves do not all pass through a single point upon closer inspection. The right plot shows the mean (which does not change) and skewness as *r* is increased from 0 to 1. Changes in *r* were obtained by starting with the parameter set: $\rho = 1/120$, $\gamma = 0.2$, $\eta = 2$, $\theta = 1$, and then raising $\rho$ in increments of 1/120. The remaining dimensionless parameters were adjusted so that Eq. (39) holds for all parameter sets used. The cases corresponding to $\rho = 1/120$, 26/120, 51/120, 76/120 and 101/120 are used for the left plot

By contrast, the equilibrium lipid distribution, $m^\star (a)$, depends sensitively on the relative contribution of proliferation and recruitment. Consider Fig. 9, in which we present plots of $m^\star (a)$ subject to the constraints outlined in the above paragraphs. The plots are labelled using the quantity, *r*, which is the proportion of macrophages that are sourced from proliferation, as opposed to recruitment. A simple expression for *r* can be found by taking the ratio of the total proliferation rate to the combined rate of proliferation and recruitment:

41 $$\begin{aligned} r = \frac{\rho M^\star }{\rho M^\star + (A_M^\star - M^\star )/(\kappa + A_M^\star - M^\star )} = \frac{\rho }{1 + \gamma } < 1. \end{aligned}$$

The substantial simplification shown in Eq. (41) is found by substituting the result for $A_M^\star$ in Eq. (27). We note that although $m^\star (a)$ changes with *r*, the alterations are anchored by the constraint that the average lipid per macrophage, $\mu = A_M^\star /M^\star$, is fixed. The changes therefore manifest in the higher order moments, affecting the skewness. Specifically, the skewness $\tilde{\mu }_3$ decreases monotonically as *r* increases from 0 to 1. This decrease is slight and the trend appears to be convex, indicating that changes to the statistical properties of $m^\star (a)$ decrease in magnitude as $r \rightarrow 1$. We note also that the ordering of the distributions $m^\star (a)$ swaps twice as *a* is increased: once near $a \approx 3$ (however the curves do not exactly coincide), and again near $a \approx 16$. Hence increases in *r* increase the number of macrophages with intermediate lipid loads relative to macrophages with small lipid burdens ($a \approx 1$) and heavily laden macrophages ($a \gg 1$).

## Discussion

In this paper we present a differential equation population model for macrophage lipid accumulation and necrotic core formation in an atherosclerotic plaque. The defining feature of this model is the use of a structural variable *a* which represents the lipid contained in macrophages and apoptotic cells, including the endogenous lipid in cell membranes. From this structured integro-PDE model we are also able to derive an ODE model for the total population of macrophages and apoptotic cells and the lipid that those populations contain.

Our model represents macrophage proliferation in a differential equation model for plaque growth for the first time. Although macrophage proliferation is known to be a key event in atherosclerosis (Robbins et al. 2013), the role of macrophage proliferation in plaque development is not well understood. Proliferation introduces nonlocal terms into the partial integro-differential equations first formulated in Ford et al. (2019). Our model equations therefore have commonalities with pantograph-type equations used to model cell proliferation in other contexts (see, for example Hall and Wake 1989; Efendiev et al. 2018). The model in this paper is more complicated than models with cell populations only, as we are representing, not only cells, but also the lipid loads that they carry. Nevertheless we show that a steady state solution for the PDE can be derived analytically in the special case where there is no efferocytosis (Appendix C).

Numerical solutions indicate that the model plaque transitions through multiple distinct stages prior to reaching a steady state (see Sect. 3.1). Interestingly, the results suggest that much of the necrotic core growth occurs while the number of live and apoptotic macrophage populations are rising very slowly (see $10 \le t \le 40$ in Fig. 2). Hence the number of live macrophages may be a poor indicator of early plaque progression when considered on its own. These observations are also consistent with the work of Lui and Myerscough (2021), and are probably due to preferential uptake of apoptotic lipid over necrotic lipid by live macrophages (the assumption $\theta < \eta$ in our model).

The fate of the model plaque and the lipid profile of the population of macrophages in the plaque are determined by the parameters $\rho$ (rate of macrophage proliferation relative to apoptosis), $\gamma$ (rate of macrophage emigration relative to apoptosis), $\eta$ (dimensionless efferocytosis rate) and $\theta$ (dimensionless uptake rate of necrotic lipid). We find that the model tends to a steady state provided that $\rho < 1 + \gamma$, typically doing so within $t = 200$ macrophage lifetimes. The case $\rho \ge 1 + \gamma$ corresponds to the unphysical scenario in which macrophages proliferate faster than they leave the system via apoptosis or emigration, and is not considered in our analysis. This reflects our assumption of linear growth dynamics, and this unphysical behaviour may be suppressed by a more sophisticated model that accounts for tissue crowding. We find that the steady state is unique when it exists, and numerical solutions indicate that it is stable.

The ODE steady state results show that increases in the proliferation rate produce increases in the number of live macrophages at steady state, $M^\star$, and decreases in eventual necrotic core lipid content, $N^\star$. The result that $M^\star$ increases with $\rho$ is unsurprising and consistent with the experimental results of Tang et al. (2015) which show that a reduction in macrophage proliferation in mice does, indeed, reduce the number of plaque macrophages. The reduction in $N^\star$ predicted by the model is primarily due to the increase in overall uptake rate of necrotic lipid in the plaque, which is proportional to $\theta M^\star$. However when the efferocytosis rate is sufficiently high ($\eta > \frac{(1+\gamma ) \nu }{\lambda }$), increases in the proliferation rate also produce decreases in the amount of apoptotic lipid in the plaque. Hence the decrease in necrotic core lipid content in this regime is also partially attributable to the rapid ingestion of apoptotic cells, and the corresponding decrease in secondary necrosis occurring within the plaque.

The effects of proliferation are modulated by changes in the other parameters of the model. We find that the efferocytosis parameter $\eta$ has a particularly significant effect. When $\eta$ is low, increasing the rate of proliferation makes very little difference to the necrotic core lipid content until proliferation rates are close to the upper limit $1 + \gamma$; a large number of plaque macrophages produces no benefit if these macrophages are ineffective at efferocytosis. Poor efferocytosis is known to be a feature of vulnerable plaques (Tabas 2010) and this modelling suggests that increasing proliferation is unlikely to be a good therapeutic intervention unless macrophages are also effectively removing apoptotic cells and necrotic material. If efferocytosis is defective, proliferation merely introduces more lipid into the plaque macrophage population and will therefore increase the lipid in apoptotic cells and thence, via secondary necrosis, the amount of lipid in the necrotic core.

Emigration, which increases with $\gamma$, appears to have a mixed effect on necrotic core lipid content. For proliferation rates $\rho < 1$, increases in $\gamma$ decrease core size until $\gamma \approx 0.15$ and increase core size thereafter. This trend probably occurs because plaques with low emigration rates contain macrophages with a higher lipid burden. When these lipid-laden macrophages emigrate from the lesion their internalised lipid is also removed from the system and so does not contribute to the necrotic core. If instead the emigration is high, macrophages leave the plaque before they can phagocytose significant amounts of necrotic lipid, and so the core grows due to lack of necrotic lipid uptake. For proliferation rates $\rho \ge 1$, the results indicate that increases in $\gamma$ monotonically increase $N^\star$. Hence, the model predicts that increasing the migratory propensity of a self-replenishing $(\rho \ge 1)$ macrophage population will always increase necrotic core lipid content.

Steady state analysis of the full model indicates that proliferation contributes to an overall decrease in macrophage lipid burden. The resulting macrophage lipid distribution, $m^\star (a)$, becomes increasingly peaked at lower values of *a* with a correspondingly lower proportion of macrophages with high lipid loads as $\rho$ is increased. Given that proliferation is lower in early stage plaque than in late stage plaque in mice ( Robbins et al. 2013), if the model is correct, then we would expect the ratio of relatively unladen macrophages (say $1< a < 3$) to heavily laden macrophages (say $a > 10$) to be higher in late stage plaques than in early plaques.

The results indicate that $m^\star (a)$ can adopt four qualitatively distinct profiles. Profiles (a) and (b) in Fig. 7 are common to this study and Ford et al. (2019). Profiles (c) and (d), which occur for $\rho$ large enough, are unique to the current study and are characterised by the global maximum occurring at some $a = a_\text {max}>1$. We note that profiles (a)-(d) can be resolved by varying only the rates of proliferation ($\rho$) and efferocytosis ($\eta$), which are the two non-local processes affecting lipid load in this model. Our results therefore highlight the potency of non-local terms in determining the qualitative features of solutions to differential equations.

The results of the model presented here, as with all conceptual models, need to be interpreted carefully and with the model assumptions in mind. In particular, this model is built on the assumption that macrophage behaviour is not dependent on lipid load. Experimental observations suggest that emigration and proliferation, for example, both depend on a cell’s lipid load ( Pataki et al. 1992; Kim et al. 2018). Lipid-dependent macrophage behaviour is the focus of the study by Watson et al. (2023). Experimental observations also indicate that macrophages have a finite capacity for phagocytosis due to both physical and metabolic constraints ( Zent and Elliott 2017). The finite lipid capacity of macrophages is a focus of the model by Chambers et al. (2023).

This model, notably, does not contain any mechanism for resolving plaque growth or for plaque regression. To include such behaviour in the model requires either carefully designed functions for lipid dependent behaviour or the introduction of other terms or other species (such as M2 macrophages) into the model ( Tabas and Bornfeldt (2016)).

In conclusion, we present here a model for populations of live macrophages that proliferate and apoptotic macrophages in an atherosclerotic plaque where the model populations are structured by the amount of lipid each cell contains. We show that in the model, macrophage proliferation generally increases the number of macrophages and increases the proportion of plaque lipids that are inside macrophages, rather than in the necrotic core. In plaques where efferocytosis is effective, macrophage proliferation can significantly reduce the size of the necrotic core. In a proliferative population of plaque macrophages there will be fewer macrophages with very high lipid loads, with macrophages predominantly attaining a small but non-zero lipid burden. By splitting lipid loads when heavily laden macrophages (foam cells) divide, proliferation spreads the macrophage lipid burden more evenly across the population.

## Figures and Tables

**Fig. 10:**
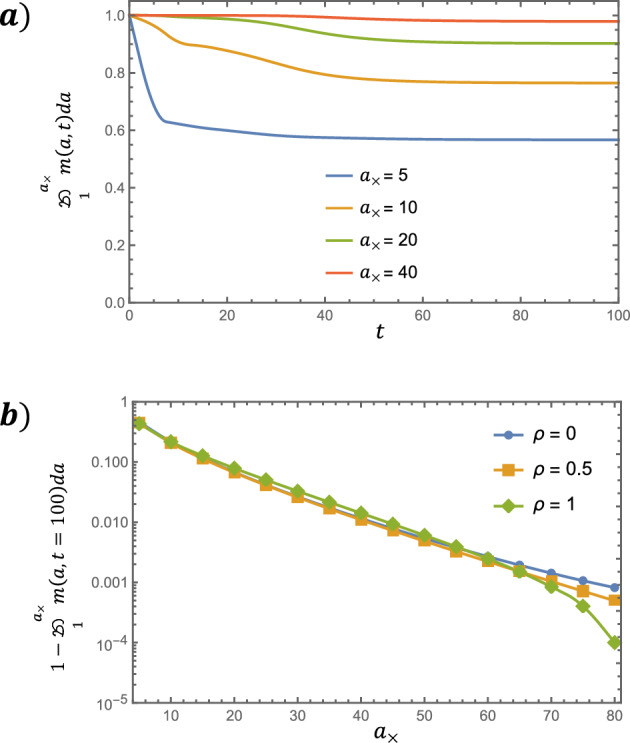
Quantifying the error in *m*(*a*, *t*) in numerical solutions of Eqs. (18)–(26). Plot a) shows how omission of the proliferation source term for $a > (a_\times + 1)/2$ causes a loss in area under the curve with time *t*. This integral is approximated via the trapezoidal rule. Larger lipid domain sizes, $a_\times$, yield smaller deviations from the mathematically correct value of 1. Parameter values for **a** are set to $\rho = 0.5$, $\eta = 2$, $\gamma = 0.25$ and $\theta = 0.4$. Plot b) shows the deviation of the area under the curve from unity at $t = 100$ (near steady state). Note the logarithmic scale. Parameter values for **b** are set to $\eta = 2$, $\gamma = 0.25$ and $\theta = 0.4$

## Appendix A: Numerical Error of Omitting the Proliferation Source

See Fig. 10.

## Appendix B: ODE Equilibrium Trends

Here we provide a proof of the trends mentioned in Sect. 3.2.1 by explicitly considering partial derivatives with respect to the proliferation parameter, $\rho$.

- $$M^\star $$:
  We note that the solution (29) can be written as:
  B.1 $$\begin{aligned} M^\star = \frac{-k_1 + \sqrt{k_1^2 + 4k_2}}{2}, \end{aligned}$$
  where
  B.2 $$\begin{aligned} k_1&= \frac{\lambda - 1 + \gamma (\kappa + \gamma \kappa + \lambda ) - \rho (\lambda + \gamma \kappa )}{1 + \gamma - \rho },&k_2&= \frac{\lambda }{1 + \gamma - \rho }. \end{aligned}$$
  It follows from the chain rule that:
  B.3 $$\begin{aligned} \frac{\partial M^\star }{\partial \rho }&= \frac{\partial M^\star }{\partial k_1} \frac{\partial k_1}{\partial \rho } + \frac{\partial M^\star }{ \partial k_2} \frac{\partial k_2}{\partial \rho }, \nonumber \\&= \frac{1}{2 (1 + \gamma - \rho )^2} \Big ( \frac{k_1}{\sqrt{k_1^2 + 4k_2}} - 1 \Big ) + \frac{\lambda }{(1 + \gamma - \rho )^2} \frac{1}{\sqrt{k1^2 + 4k_2}}. \end{aligned}$$
  Since $k_2 > 0$ (using that $\rho < 1 + \gamma$ as noted in Sect. 2.5), the first term is positive and hence also $\frac{\partial M^\star }{\partial \rho } > 0$.
- $$P^\star $$:
  From the relation for $P^\star$ in (27), we note that $P^\star$ is an increasing function of $M^\star$. From the result proved above, we also have $\frac{\partial P^\star }{\partial \rho }$.
- $$A_M^\star $$:
  From the relation for $A_M^\star$ in (27), we can write:
  B.4 $$\begin{aligned} A_M^\star - M^\star = \kappa \Big ( \frac{1}{1+ (\rho - 1 - \gamma ) M^\star } - 1 \Big ). \end{aligned}$$
  Differentiating the above line with respect to $\rho$ gives:
  B.5 $$\begin{aligned} \frac{\partial A_M^\star }{\partial \rho } - \frac{\partial M^\star }{\partial \rho }&= \kappa \frac{1 + \gamma - \rho }{(1 + M^\star [1 + \gamma - \rho ]^2)} \frac{\partial M^\star }{\partial \rho }. \end{aligned}$$
  Using that $\frac{\partial M^\star }{\partial \rho } > 0$, we find that $\frac{\partial A_M^\star }{\partial \rho }> \frac{\partial M^\star }{\partial \rho } > 0$.
- $$N^\star $$:
  The relation for $N^\star$ in (27) can be written as:
  B.6 $$\begin{aligned} N^\star = \frac{\nu }{\theta } \frac{A_P^\star }{A_M^\star } \cdot \frac{A_M^\star }{M^\star }. \end{aligned}$$
  The fraction $\frac{A_P^\star }{A_M^\star }$ is a decreasing function of $M^\star$, using the relation for $A_P^\star$ in (27). Hence $\frac{A_P^\star }{A_M^\star }$ is also a decreasing function of $\rho$, using that $\frac{\partial M^\star }{\partial \rho } > 0$. The fraction $\frac{A_M^\star }{M^\star }$ can be written as
  B.7 $$\begin{aligned} \frac{A_M^\star }{M^\star } = 1 + \frac{\kappa }{\frac{1}{1 + \gamma - \rho } - M^\star }. \end{aligned}$$
  The denominator, $\frac{1}{1 + \gamma - \rho } - M^\star$, can be shown to be an increasing function of $\rho$ using (B.3). Hence, $\frac{\partial }{\partial \rho } (A_M^\star / M^\star ) < 0$. It then follows that $\frac{\partial N^\star }{\partial \rho } < 0$ since $N^\star$ is the product of two positive and decreasing functions of $\rho$.
- $$A_P^\star $$:
  By differentiating the expression for $A_P^\star$ in (27) using the chain rule, we can write:
  B.8 $$\begin{aligned} \frac{\partial A_P^\star }{ \partial \rho }&= \frac{\partial A_P^\star }{\partial A_M^\star } \frac{\partial A_M^\star }{\partial \rho } + \frac{\partial A_P^\star }{\partial M^\star } \frac{\partial M^\star }{\partial \rho }, \nonumber \\&= \frac{1}{\eta M^\star + \nu } \Big ( \frac{\partial A_M^\star }{\partial \rho } - \eta A_P^\star \frac{\partial M^\star }{\partial \rho } \Big ). \end{aligned}$$
  By analysing the sign of the bracketed term, we find that $\frac{\partial A_P^\star }{\partial \rho } > 0$ when $\eta < \frac{(1 + \gamma ) \nu }{\lambda }$ and $\frac{\partial A_P^\star }{\partial \rho } < 0$ if $\eta > \frac{(1 + \gamma ) \nu }{\lambda }$. The singular case, $\eta = \frac{(1+ \gamma ) \nu }{\lambda }$ gives $\frac{\partial A_P^\star }{\partial \rho } = 0$.

## Appendix C: Analytical Solution for $m^\star (a)$ when $k_1 = 0$

Taking the Laplace transform of Eq. (31) gives the functional algebraic relation:

C.9 $$\begin{aligned} s \hat{m}(s) - m^\star _1 = k_1 e^{-s} \hat{m}(s)^2 - k_2 \hat{m}(s) + \frac{k_3}{2} \hat{m}(s/2), \end{aligned}$$

where

C.10 $$\begin{aligned} \hat{m}(s) := \int _0^\infty m^\star (a+1) e^{-as} da. \end{aligned}$$

Solving Eq. (C.9) when $k_1 = 0$ gives:

C.11 $$\begin{aligned} \hat{m}(s) = \frac{m^\star _1}{s + k_2} + \frac{k_3}{2 (s + k_2)} \hat{m}(s/2). \end{aligned}$$

Equation (C.11) can be solved by iteration. Assuming that Re$(s) > 0$, recursive application of Eq. (C.11) gives

C.12 $$\begin{aligned} \hat{m}(s)&= \frac{m^\star _1}{s + k_2} + \frac{k_3}{2 (s + k_2)} \hat{m}(s/2) \nonumber \\&= \frac{m^\star _1}{s + k_2} + \frac{k_3}{2 (s + k_2)} \Big ( \frac{m^\star _1}{s/2 + k_2} + \frac{k_3}{2 (s/2 + k_2)} \hat{m}(s/4) \Big ) \nonumber \\&\, \, \, \vdots \nonumber \\&= m^\star _1 \sum _{j = 0}^n \frac{(k_3/2)^j}{\prod _{\ell =0}^j (s/2^\ell + k_2)} + \frac{(k_3/2k_2)^{n+1}}{\prod _{j=0}^n (1 + k_2 s/2^j)} \hat{m}(s/2^{n+1}), \end{aligned}$$

for every $n \ge 0$. We note that the second term tends to zero as $n \rightarrow \infty$. To see this, recall first that $\hat{m}(0) = 1$ from the non-dimensionalisation (16). Substituting $s = 0$ into Eq. (C.11) gives $k_3/(2k_2) = 1 - m^\star _1/k_2 < 1$. It then follows from our assumption that Re$(s) > 0$ and the continuity of $\hat{m}$ at $s = 0$ that

C.13 $$\begin{aligned} \Bigg \vert \frac{(k_3/2k_2)^{n+1}}{\prod _{j=0}^n (1 + k_2 s/2^j)} \hat{m}(s/2^{n+1}) \Bigg \vert \le \Big ( \frac{k_3}{2k_2} \Big )^{n+1} \vert \hat{m}(s/2^{n+1}) \vert \rightarrow 0 \end{aligned}$$

as $$n \rightarrow \infty $$. Hence,

C.14 $$\begin{aligned} \hat{m}(s) = m^\star _1 \sum _{j = 0}^n \frac{(k_3/2)^j}{\prod _{\ell =0}^j (s/2^\ell + k_2)}. \end{aligned}$$

The product in Eq. (C.14) can be decomposed into a sum of partial fractions. Doing so allows us to write

C.15 $$\begin{aligned} \hat{m}(s) = \frac{m^\star _1}{k_2} \sum _{j=0}^\infty \Big ( \frac{k_3}{2k_2} \Big )^j \sum _{i=0}^j \frac{1}{\prod _{\ell = 0, \ell \ne i}^j (1-2^{i-\ell })} \frac{1}{1 + k_2 s/2^i}. \end{aligned}$$

Finally, we take the inverse Laplace transform term-by-term (justified by the uniform convergence of the above series) to obtain the solution (35).
